# The Presence of *Dirofilaria immitis* in Domestic Dogs on San Cristobal Island, Galapagos

**DOI:** 10.3390/pathogens11111287

**Published:** 2022-11-02

**Authors:** Carla Andreea Culda, Romane Dionnet, Andra Celia Barbu, Andrada Silvia Cârstolovean, Teodora Dan, Jaime Grijalva, Priscilla Espin, Rommel Lenin Vinueza, Marylin Cruz, Diego Páez-Rosas, Leon Renato, Andrei Daniel Mihalca

**Affiliations:** 1 Department of Parasitology and Parasitic Diseases, University of Agricultural Sciences and Veterinary Medicine of Cluj-Napoca, 400372 Cluj-Napoca, Romania; 2 Escuela de Medicina Veterinaria, Universidad San Francisco de Quito, Cumbayá, Quito 150157, Ecuador; 3 Agencia de Regulación y Control de la Bioseguridad y Cuarentena para Galápagos, Isla San Cristóbal 200152, Galápagos, Ecuador; 4 Laboratorio de Entomología Médica & Medicina Tropical LEMMT, Universidad San Francisco de Quito, Cumbayá, Quito 150157, Ecuador; 5 Galapagos Science Center, Universidad San Francisco de Quito, Isla San Cristóbal 200150, Islas Galápagos, Ecuador; 6 Dirección del Parque Nacional Galápagos, Unidad Técnica Operativa San Cristóbal, Isla San Cristóbal 200150, Islas Galápagos, Ecuador

**Keywords:** *Dirofilaria immitis*, domestic dogs, canine heartworm disease, zoonoses, San Cristobal Island

## Abstract

This study's concept was outlined around the principle of conservation medicine in a biodiversity hotspot from the Neotropical realm: the Galapagos Islands. The wildlife balance has been modified by multi-host parasites introduced with some domestic animals (dogs and cats). The endemic and endangered species, the Galapagos sea lion (GSL, *Zalophus wollebaeki*), has been exposed to pathogens of canine and feline origin that could become a significant conservation problem for this species. One of these potential cases is the filarial heartworm infection, *Dirofilaria immitis*, which has been reported on other pinnipeds, with fatalities and clinical symptoms. Therefore, this study evaluated the presence of the microfilaria of *D. immitis* in dogs from Puerto Baquerizo Moreno, San Cristobal Island, where the largest rookery of GSLs lives and where the proximity to domestic dogs is the most intimate compared to other rookeries of the archipelago. Between July and September 2021, 587 blood samples were collected from owned dogs of Puerto Baquerizo Moreno. Overall, 10 dogs (1.7%) were positive for the presence of the microfilaria of *D. immitis* with a confidence interval of 0.7–2.8%. No other filarial species were identified. Significant differences in prevalence between different dog categories were observed only for the age (*p* = 0.001). This study represents the first report of *D. immitis*, the agent of canine heartworm disease, in dogs from San Cristobal Island. Hence, the presence of the microfilaria of *D. immitis* in the blood of dogs could increase the risk of infection to which the GSL is exposed in the region.

## 1. Introduction

**Figure 1:**
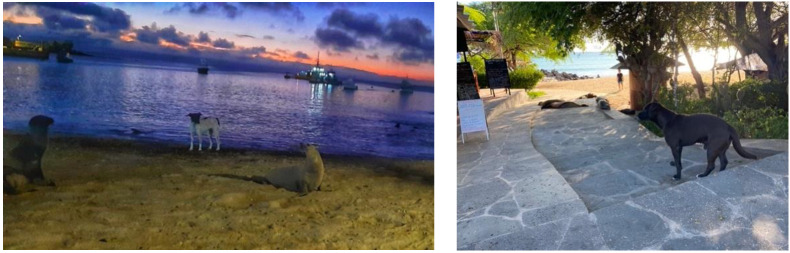
Close contact between dogs and Galapagos sea lions.

The Galapagos Islands are located in the Eastern Tropical Pacific, about 906 km from the coast of mainland Ecuador, and have been named a “natural laboratory of evolution” [1,2,3,4]. Approximately, 80% of the land birds, 97% of the reptiles and land mammals, and more than 30% of the plants are endemic and unique to the Galapagos [5]. Despite all the conservation efforts to maintain this biodiversity, 23 animal species are facing extinction or have disappeared already [6]. The main factors endangering the endemic species of Galapagos are habitat loss and/or fragmentation, introduced predators, disease agents, and competitors, human activity, including increased tourism, waste disposal, climate change and El Niño events [6,7,8,9,10]. Among the iconic species of the islands is the endemic Galapagos sea lion (GSL), *Zalophus wollebaeki*. Due to increased climate variability (i.e., ENSO event) and anthropogenic impacts (e.g., fisheries and introduced species), a drastic decrease in the population of the GSL has been observed and the species is currently listed as endangered by IUCN [10,11]. Another factor negatively impacting GSL populations is the interaction with domestic animals [9,12,13]. This interaction is particularly significant in rookeries living close to human settlements. Puerto Baquerizo Moreno, located on San Cristobal Island, is home to “El Malecon” rookery, which contains the largest population of GSL in the entire archipelago [10,14]. Here, GSLs are found close to human settlements and in close contact with domestic dogs, which increases the risk of pathogen spillover (Figure 1). However, mosquitoes are able to travel relatively long distances between their infection with L1 of *D. immitis* and the time when the larvae become infective, L3 [15,16].

These conditions have been linked to the identification of various pathogens of canine origin in GSLs, such as canine distemper virus, *Leptospira*, *Mycoplasma* spp. and various intestinal parasites [13,17,18]. Moreover, parvoviruses, herpesviruses, caliciviruses, *Toxoplasma* or *Brucella* could also pose a threat to this species [19,20,21]. Most of these potential pathogens in GSLs have been identified in local canine populations in the Galapagos Islands [22]. Additionally, cats from the Galapagos Islands have been reported to be infected with several of these pathogens [22]. To highlight the importance of canine and feline pathogens from a conservation medicine perspective, we summarize the findings of these studies in Table 1. The parasites of dogs have been investigated on a few occasions in the Galapagos Islands: Isabela [22,23], Santa Cruz [23,24,25,26], San Cristobal [23] and Floreana [27]. These studies revealed a high diversity of parasitic and vector-borne pathogens, including *D. immitis* and *Leishmania donovani*, and also various flea-borne and tick-borne bacteria (Table 1).

Of particular interest is the filarial heartworm, *D. immitis*, reported prior to the current study with a concerning prevalence of antigens on Isabela Island (34%) in 95 dogs tested [22] and Santa Cruz Island (6.9%) in 58 dogs tested [26]. Additionally, an older study reported that 77% of dogs and 18% of cats on Floreana Island had antibodies against *D. immitis* [27]. However, none of these studies evaluated the presence of microfilaria, hence the reservoir role of domestic carnivores remains unknown. Since, the presence of *D. immitis* has not been investigated in dogs that inhabit San Cristobal Island, where the largest GSL rookery resides [10,14], it is particularly important to define the potential impact of this disease in dogs and its possible transmission to the endemic fauna. The canine heartworm is transmitted between mammal hosts by mosquitoes of various genera. In Galapagos, three species of mosquitoes have been reported (all introduced): *Aedes aegypti*, *Aedes taeniorhynchus* and *Culex quinquefasciatus* [26,27,28,29,30], all known to be competent vectors for *D. immitis* [31,32].

*Dirofilaria immitis* is often responsible for severe cardiac and respiratory clinical signs and death in dogs and cats. Concerningly, *D. immitis* has been reported in various pinnipeds such as the hooded seal (*Cystophora cristata*) [33], captive common seals (*Phoca vitulina*) in Canada, Portugal [34] and Korea [35], African fur seals (*Arctocephalus pusillus*) in an oceanographic park in Portugal [34] and California sea lions (*Zalophus californianus*) in zoological parks in Florida [36], Louisiana [37] and Japan [38] with fatalities and clinical signs such as cardiopulmonary impairment, coughing and labored breathing [34,35,36,37,38,39]. Even though the presence of *D. immitis* has been documented in the Galapagos Archipelago [22,23,24,25,26,27], several crucial gaps concerning the canine heartworm remain; most importantly, the risk posed to the endemic and endangered GSL, which live on all of the islands and are in close contact with dogs and mosquitoes. Therefore, the aim of this study was to evaluate the presence of the microfilaria of *D. immitis* in dogs from San Cristobal Island where the contact of GSLs with domestic dogs is the most intimate.

## 2. Materials and Methods

### 2.1. Sample Collection

**Figure 2:**
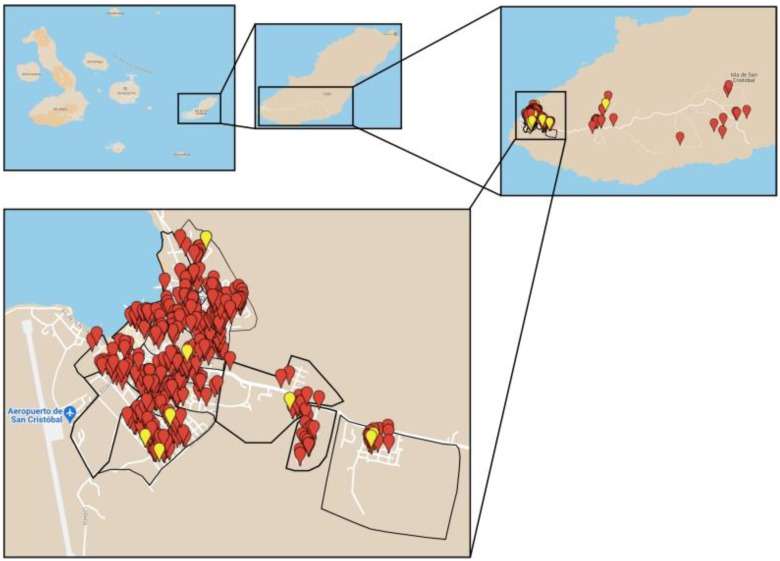
Distribution of sampled and positive dogs. A detailed interactive map is available as Supplementary Materials here (https://www.google.com/maps/d/edit?mid=1IfYrPtQGO455j76RSZust5gkdF80hCML&usp=sharing). Red pins represent the negative dogs and yellow pins represent the positive dogs.

Between July and September 2021, 587 blood samples (532 samples screened in the urban area and 55 samples screened in the rural area) were collected from owned dogs (311 males and 276 females) from all 15 neighborhoods of Puerto Baquerizo Moreno (defined as urban) and from rural areas (Figure 2). The sampling strategy consisted of the random selection of blocks of urban and rural neighborhoods. The number of dogs to be selected for blood testing was based on the human population data obtained from the municipality of San Cristobal Island. The observed human–dog ratio on San Cristobal Island was 6:1, which extrapolated to 600 dogs [40].

The minimum sample size was calculated for San Cristobal Island based on an estimated population size [41], the expected prevalence and 95% confidence level (http://www.winepi.net/f101.php, accessed on 21 October 2022 [42]) (Table 2).

The blood collection was performed through a door-to-door approach. Each owner was informed about the importance for the ecosystem of this study through a verbal explanation. For the collection of blood, all owners provided written consent. The animals were restrained manually with the help of a trained assistant and the owner of the dog. Blood was collected from the cephalic vein of the foreleg or the saphenous vein using S-Monovette EDTA K_2_E tubes. A total volume of 3–5 mL of blood was collected from each animal. At the end of the procedure, all participants received a flyer with a short description of the heartworm disease. Additionally, for each dog, a questionnaire was completed with data concerning the name, age, sex (including the neutered/non-neutered status), breed, type of housing, travel history, previous antiparasitic treatments and vaccinations, medical history, clinical signs at the time of veterinary evaluation. For each dog, the microchip number was recorded, together with the date of the blood collection. Dogs without a microchip were provided with one with the agreement of Galapagos Biosecurity Agency (ABG). Only dogs that were at least 6 months old were included in the study, as the prepatency for *D. immitis* is usually 6–7 months [43,44].

### 2.2. Blood Examination

The examination of the blood was conducted at the Galapagos Science Center (GSC) facility of Universidad San Francisco de Quito (USFQ) in San Cristobal Island. All tubes with blood samples collected were stored at 4 °C until examination, for no longer than 12 h. For detection of the microfilariae, the Knott’s test was used [45,46,47,48]. The modified Knott’s test is a concentration method that aims to detect microfilariae species in blood samples [44]. It is a method that is more sensitive than the standard blood smear as it concentrates the microfilariae from a relatively large volume of blood (1 mL). In addition to serologic tests (which detects antigens of adult females), it shows that the dog is still infective to mosquitoes and the infection is patent. The microfilaria of *D. immitis* was identified based on a morphological differentiation of the character of the species (length of the cephalic space, anterior end to the last body nucleus and the length of the portion of the tail without nuclei) [49,50].

### 2.3. Statistical Analysis

All statistical analysis was performed using the EpiTools software. Owned dogs were grouped by age, as follows: <1 year, 1–4 years, 4–10 years, >10 years, unknown. Age groups were formed based on the possibility of infection given the time of exposure to possible vectors for each dog. Odds ratios (ORs), 95% confidence intervals (CI) and *p*-values were calculated for statistically different prevalence by univariate logistic regression. The criterion for statistical significance was *p* ≤ 0.05. Binomial proportion 95% CI were calculated for positive dogs.

## 3. Results

Overall, out of 587 tested dogs, 10 were positive for the presence of the microfilaria of *D. immitis* (1.7%, 95% CI: 0.7–2.8). No other filarial species were identified, and none of the positive dogs presented clinical signs characteristic of *D. immitis* infection. The population of dogs ranged in age from 6 months to 15 years with a median age of 47 months, and included both non-neutered (190 males, 109 females) and neutered (113 males, 156 females). The prevalence across the different categories is shown in Table 3.

Significant differences for the prevalence between different dog categories were observed only for age (*p* = 0.001), with a higher risk in dogs aged between 4 and 10 years. No statistical differences were recorded for the sex (*p* = NS), breed (*p* = NS), type of housing (*p* = NS) and environment (*p* = NS), while the prevalence by neighborhood in the town of Puerto Baquerizo Moreno is shown in Table 4. No significant association was found between neighborhoods and the prevalence of *D. immitis* (*p* = NS).

## 4. Discussion

This study represents the first report to demonstrate the direct presence of the microfilaria of *D. immitis* in the blood of dogs in the Galapagos Archipelago, as previous studies targeted the detection of antibodies [27], antigens [22,23,24,25,26] or DNA [26]. The presence of an endemic cycle for *D. immitis* depends on the presence of suitable definitive hosts (dogs), vectors (mosquitoes) and the nematodes. The presence and abundance of mosquitoes and the development of *D. immitis* larvae in mosquitoes are dependent on climatic factors, the most important being the temperature and availability of mosquito breeding sites [51,52]. Hence, climate and weather have a significant impact on the prevalence of canine heartworm. *Dirofilaria immitis* L1 larvae need an average temperature higher than 15 °C to develop to L3 in the mosquitoes [53]. Additionally, a recent study demonstrated that cumulative exposure to adequate temperatures can result in the progression of larvae from microfilaria to the L3 infective stage [54]. From this point of view, the Galapagos Archipelago represents a suitable biotope for the development of the mosquito vector and of the *D. immitis* larvae [55]. Furthermore, sea lions spend more time on land [56], especially in the evening when mosquitoes are active [57].

In the current study, the prevalence of heartworm microfilaremia in dogs (1.7%) was lower than the antigen seroprevalence found in the other islands of the Galapagos Archipelago such as Santa Cruz (6.9%) [26] and Isabela (34%) [22]. Similar studies on other oceanic islands showed a generally higher prevalence: Hawaii (0.7–1.5%) [58], Gran Canaria (12%) [59] and Tenerife (20–34%) [59,60]. Although the climatic conditions on oceanic islands can significantly differ, even over short distances (i.e., coastal vs. highlands), it seems that these are not enough to influence the infection prevalence, as previously demonstrated [60]. Previous studies showed that the prevalence of *D. immitis* seems to be higher in older dogs [59,60,61], which is consistent with our results. This is associated with the longevity of patent *D. immitis* infections in dogs, which may be up to 5–7 years [62,63]. Although the prevalence of *D. immitis* microfilaria in our study was higher in dogs housed outdoors, no statistical significance was found in our study. However, previous studies consider this a risk factor [60,61].

Although the Knott’s test has been shown to be less sensitive than the antigen test [51,52], the method has several advantages: (i) it allows the detection of other filarial species; (ii) it is more specific; (iii) it is cheaper; and (iv) it directly demonstrates the reservoir role of dogs, which was in fact our main rationale for this study. However, antigen detection-based methods can also detect occult heartworm infection even if microfilariae are not present in the blood [53,61,64,65].

Given that: (i) positive dogs were found in the urban environment of the town of Puerto Baquerizo Moreno; (ii) the largest colony of the endangered GSL lives in close vicinity with dogs (closest positive dogs were 350 m away from the GSL colony, which represent a higher risk of infection to mosquitoes that could subsequently feed on sea lions); (iii) dogs are often roaming free in the very close vicinity of GSL; (iv) three mosquito species that have a demonstrated vectorial competence for *D. immitis* are reported in the town; (v) pinnipeds were previously demonstrated as suitable hosts for *D. immitis* and can develop clinical signs, we consider that GSLs are exposed to the risk of infection.

Hence, measures to limit the risk of transmission to other dogs, humans or GSLs should be taken, such as: the strict control of dog movement, routine surveillance of dogs for the detection of *D. immitis*, treatment of positive dogs, implementation of a strategic control program with periodic administration of macrocyclic lactones to all dogs on the island and use of repellent collars on dogs for the prevention of mosquito bites and environmental control of mosquitoes. However, the antiparasitic treatments received by dogs in the Galapagos Islands are very scarce. This is a worrying situation given that antiparasitic treatment in these animals varies over time from a few months to years (Mihalca and Culda, unpublished data). Therefore, treatment campaigns should be carried out for a certain period of time targeting the elimination of the infection from domestic dogs from the island followed by more strict measures to prevent re-introduction

In addition, a neglected reservoir can be represented by cats. These were introduced to the archipelago and they have a negative impact on wildlife [23,55]. Moreover, the majority of cats roam free in the environment and they have a demonstrated role as reservoirs for *D. immitis* [66,67,68,69].

Although cats are less competent reservoirs than dogs, their role should not be neglected and we recommend their inclusion in future surveillance and control programs for *D. immitis*.

## 5. Conclusions

The main scientific impact of the study is that it demonstrates the presence of infective *D. immitis* larvae in domestic dogs, which, due to their physical proximity to sea lions, could represent a potential infection source. The future perspectives for the research are: (i) to sample the other islands (Isabela, Floreana and Santa Cruz) to complete the data on the prevalence and distribution of *D. immitis* across the Galapagos Archipelago; (ii) to understand the transmission cycle of *D. immitis* and identify the most important mosquito vector species; (iii) to evaluate the mosquito blood source around urban and wild rookeries of GSL; (iv) to investigate the possible role of GSLs as reservoirs for *D. immitis.* Hence, understanding the risk of such an exposure is crucial for future GSL conservation plans and actions.

## 6. Ethics

This study received approval from the commission for Bioethics within the University of Agricultural Sciences and Veterinary Medicine of Cluj-Napoca (USAMV Cluj-Napoca, Romania) code: 233, and a permit from the Institutional Animal Care and Use Committee (IACUC) (Comité de Ética en el Uso de Animales en Investigación y Docencia de la USFQ); code: 006. Fieldwork at the Galapagos was performed in coordination with the Galapagos Biosecurity Agency ABG under an agreement for Scientific Collaboration between USFQ and the ABG agency.

## Figures and Tables

**Table 1:** Distribution of pathogens on the Galapagos Islands.

Pathogen	Isabela	Santa Cruz	San Cristobal	Floreana
Canine distemper virus	+	+	−	−
Canine parvovirus	+	+	−	−
Canine adenovirus	+	+	−	−
Canine parainfluenza virus	+	+	−	−
*Ehrlichia* spp.	+	+	−	−
*Anaplasma* spp.	+	+	−	−
*Bartonella* spp.	+	−	−	−
*Mycoplasma haemocanis*	+	−	−	−
*Giardia* spp.	−	+	+	−
*Leishmania donovani*	+	−	−	−
*Sarcocystis canis*	+	+	−	−
*Cystoisospora* spp.	+	+	−	−
*Cryptosporidium* spp.	−	+	−	−
*Toxocara* spp.	+	+	+	−
*Ancylostoma* spp.	+	+	+	−
*Dirofilaria immitis*	+	+	−	+

+ = presence of pathogens; − = absence/non-testing of pathogens.

**Table 2:** Sample size selection and randomization.

Area	Estimated Human Population ^1^	Estimated Number of Dogs ^2^	Min. Sample Size	No. of Collected Samples
San Cristobal	7330	1222	269	587

^1^— [41]; ^2^—based on the 6:1 ratio according to [40].

**Table 3:** Prevalence of microfilariae of *D. immitis* in dogs from San Cristobal by sex, age, environment and housing, assessed by Knott’s test.

	No. of Samples	Positive (%)	OR	95% CI	*p*-Value
Total	587	10 (1.7%)		0.7–2.8%	
Sex					
Males	311	5 (1.6%)	0.8856	0.2–3.0%	1
Females	276	5 (1.8%)		0.2–3.4%	
Age					
<1 year	74	0	NA	0%	0.001 *
1–4 years	236	0		0%	
4–10 years	208	10 (4.8%)		1.9–7.8%	
>10 years	43	0		0%	
unknown	26	0		0%	
Environment					
Urban	532	8 (1.5%)	0.4046	0.5–2.6%	0.5377
Rural	55	2 (3.6%)		0–8.6%	
Housing					
Outdoor	258	8 (3.1%)	NA	1–5.2%	0.0571
Indoor	109	0		0%	
Outdoor and indoor	220	2 (0.9%)		0–2.2%	

OR—odds ratio; 95% CI—95% confidence interval; NA—not applicable; *—statistically significant.

**Table 4:** Prevalence of microfilariae of *D. immitis* in dogs from Puerto Baquerizo Moreno by neighborhood, assessed by Knott’s test.

Neighborhood (Barrio)	No. of Dogs	Positive	Prevalence (%)	95% CI	*p*-Value
Albatros	30	0	0	0	0.2136
Algarrobos	42	0	0	0	
Cactus	49	1	2%	0–6.0%	
Central	18	0	0	0	
Divino Niño	24	1	4.1%	0–12.1%	
Manatial and Isla Sur	21	1	4.7%	0–13.8%	
Estacion Terrena	89	3	3.3%	0–7.2%	
Fragatas	48	0	0	0	
Frío	8	0	0	0	
El Manzanillo	23	0	0	0	
Las Palmeras	23	2	8.7%	0–20.2%	
Peñas Altas	76	0	0	0	
Peñas Bajas	35	0	0	0	
Playa Mann	23	0	0	0	
San Francisco	23	0	0	0	

95% CI—95% confidence interval.

## Data Availability

The dataset supporting the conclusions of this study are included in the present article or in the Supplementary Materials.

## Supplementary Materials

Interactive Map_San Cristobal Island (Red pins represent the negative dogs and yellow pins represent the positive dogs, and green ones represent sea lions’ location). https://www.google.com/maps/d/edit?mid=1IfYrPtQGO455j76RSZust5gkdF80hCML&usp=sharin.
