# Correction: Culda et al. The Presence of *Dirofilaria immitis* in Domestic Dogs on San Cristobal Island, Galapagos. *Pathogens* 2022, *11*, 1287

**DOI:** 10.3390/pathogens12070856

**Published:** 2023-06-21

**Authors:** Carla Andreea Culda, Romane Dionnet, Andra Celia Barbu, Andrada Silvia Cârstolovean, Teodora Dan, Jaime Grijalva, Priscilla Espin, Rommel Lenin Vinueza, Marylin Cruz, Diego Páez-Rosas, Leon Renato, Andrei Daniel Mihalca

**Affiliations:** 1 Department of Parasitology and Parasitic Diseases, University of Agricultural Sciences and Veterinary Medicine of Cluj-Napoca, 400372 Cluj-Napoca, Romania; carla-andreea.culda@usamvcluj.ro (C.A.C.); romane.dionnet@student.usamvcluj.ro (R.D.); andra-celia.barbu@student.usamvcluj.ro (A.C.B.); andrada-silvia.carstolovean@student.usamvcluj.ro (A.S.C.); teodora.dan@student.usamvcluj.ro (T.D.); 2 Escuela de Medicina Veterinaria, Universidad San Francisco de Quito, Cumbayá, Quito 150157, Ecuador; cgrijalva@asig.com.ec (J.G.); rvinueza@usfq.edu.ec (R.L.V.); 3 Agencia de Regulación y Control de la Bioseguridad y Cuarentena para Galápagos, Isla San Cristóbal 200152, Galápagos, Ecuador; priscilla.espin@abgalapagos.gob.ec (P.E.); marilyn.cruz@abgalapagos.gob.ec (M.C.); 4 Laboratorio de Entomología Médica & Medicina Tropical LEMMT, Universidad San Francisco de Quito, Cumbayá, Quito 150157, Ecuador; rleon@usfq.edu.ec; 5 Galapagos Science Center, Universidad San Francisco de Quito, Isla San Cristóbal 200150, Islas Galápagos, Ecuador; dpaez@usfq.edu.ec; 6 Dirección del Parque Nacional Galápagos, Unidad Técnica Operativa San Cristóbal, Isla San Cristóbal 200150, Islas Galápagos, Ecuador

**Text Correction**

There was an error in the original publication [1]. In the original manuscript, we indicated that our study was the first report to demonstrate the direct presence of the microfilaria of *D. immitis* in the blood of dogs in the Galapagos Archipelago. However, we later realized that two earlier studies found microfilaria of *D. immitis* on two islands of the Galapagos Islands: Floreana and Santa Cruz.

A correction has been made to Section 4. Discussion, paragraph 1:

“This study represents the first report to demonstrate the direct presence of the microfilaria of *D. immitis* in the blood of dogs in the Galapagos Archipelago, as previous studies targeted the detection of antibodies [27], antigens [22–26] or DNA [26]. The presence of an endemic...” has been replaced with two paragraphs, as show below,

This study represents the first report to demonstrate the direct presence of circulating microfilaria of *D. immitis* in the blood of dogs in San Cristobal Island, Galapagos. Similar studies were performed in Floreana [27] and Santa Cruz [26], which assessed the detection of antibodies [27], antigens [22–26], and DNA [26].

Our study completed the previous studies [26,27] regarding the presence of circulating microfilaria of *D. immitis* in the blood of dogs in the Galapagos Archipelago. Most other studies targeted the detection of antibodies [27], antigens [22–26], or DNA [26]. The presence of an endemic cycle for *D. immitis* depends on the presence of suitable definitive hosts (dogs), vectors (mosquitoes) and the nematodes. The presence and abundance of mosquitoes and the development of *D. immitis* larvae in mosquitoes are dependent on climatic factors, the most important being the temperature and availability of mosquito breeding sites [51,52]. Hence, climate and weather have a significant impact on the prevalence of canine heartworm. *Dirofilaria immitis* L1 larvae need an average temperature higher than 15 °C to develop to L3 in the mosquitoes [53]. Additionally, a recent study demonstrated that cumulative exposure to adequate temperatures can result in the progression of larvae from microfilaria to the L3 infective stage [54]. From this point of view, the Galapagos Archipelago represents a suitable biotope for the development of the mosquito vector and of the *D. immitis* larvae [55]. Furthermore, sea lions spend more time on land [56], especially in the evening when mosquitoes are active [57].

The authors apologize for any inconvenience caused and state that the scientific conclusions are unaffected. This correction was approved by the Academic Editor. The original publication has also been updated.
